# Structural Modifications of the Brain in Acclimatization to High-Altitude

**DOI:** 10.1371/journal.pone.0011449

**Published:** 2010-07-06

**Authors:** Jiaxing Zhang, Xiaodan Yan, Jinfu Shi, Qiyong Gong, Xuchu Weng, Yijun Liu

**Affiliations:** 1 Laboratory for Higher Brain Function, Institute of Psychology, Chinese Academy of Sciences, Beijing, China; 2 Department of Physiology and Neurobiology, Medical College of Xiamen University, Xiamen, China; 3 Langone Medical Center, New York University, New York, New York, United States of America; 4 Huaxi Magnetic Resonance Research Center, West China Hospital, Sichuan University, Chendu, China; 5 Departments of Psychiatry and Neuroscience, University of Florida McKnight Brain Institute, Gainesville, Florida, United States of America; Cuban Neuroscience Center, Cuba

## Abstract

Adaptive changes in respiratory and cardiovascular responses at high altitude (HA) have been well clarified. However, the central mechanisms underlying HA acclimatization remain unclear. Using voxel-based morphometry (VBM) and diffusion tensor imaging (DTI) with fractional anisotropy (FA) calculation, we investigated 28 Han immigrant residents (17–22 yr) born and raised at HA of 2616–4200 m in Qinghai-Tibetan Plateau for at least 17 years and who currently attended college at sea-level (SL). Their family migrated from SL to HA 2–3 generations ago and has resided at HA ever since. Control subjects were matched SL residents. HA residents (vs. SL) showed decreased grey matter volume in the bilateral anterior insula, right anterior cingulate cortex, bilateral prefrontal cortex, left precentral cortex, and right lingual cortex. HA residents (vs. SL) had significantly higher FA mainly in the bilateral anterior limb of internal capsule, bilateral superior and inferior longitudinal fasciculus, corpus callosum, bilateral superior corona radiata, bilateral anterior external capsule, right posterior cingulum, and right corticospinal tract. Higher FA values in those regions were associated with decreased or unchanged radial diffusivity coinciding with no change of longitudinal diffusivity in HA vs. SL group. Conversely, HA residents had lower FA in the left optic radiation and left superior longitudinal fasciculus. Our data demonstrates that HA acclimatization is associated with brain structural modifications, including the loss of regional cortical grey matter accompanied by changes in the white matter, which may underlie the physiological adaptation of residents at HA.

## Introduction

According to WHO (1996), there were approximately 140 million people living at high altitude (HA) over 2500 m. As of 2006, approximately 12 million Tibetan natives and Han lowland immigrants permanently reside between 2200 to 5200 m on the Qinghai–Tibetan Plateau, and every year hundreds of thousands of lowlanders traveled up to the Tibetan plateau [1]. A large amount of evidences have shown these natives and immigrants in HA environment (hypoxia, hypobaric press, UV rays from the sun, cold, and dehydration) have developed adaptive changes in respiratory and cardiovascular regulations, which directly related to oxygen transport [2]–[5].

The brain is the control centre of the body. At HA, through afferent feedback, the adaptation in the cardiovascular and respiratory systems may act on the control centers in the brain. On the other hand, as the central nervous system is highly oxidative, it inevitably suffers from hypoxic stress. The mental disturbances of chronic mountain sickness may be the strongest indicator of central nervous system failing in acclimatization to HA hypoxia [6]. Several researches on HA residents mainly focused on cerebral glucose metabolic rates [7] and cerebral autoregulation [8], [9]. Up to now, how the brain of residents in structure acclimatized to HA remains unclear.

Hypoxia is one of the most important environmental factors at HA. Laboratory observations on hypoxic animals and patients suffered from chronic obstructive pulmonary diseases (COPD) or obstructive sleep apnea (OSA) revealed increases of cerebral microvessel density in the hippocampus, the cerebellum, and the motor and the somatosensory cortices [10]–[12], the impairments of grey matter (GM) in the cerebellum, the parietal cortex, the anterior cingulate, the caudate, the putamen, the thalamus, the hippocampus, and the parahippocampus [13]–[16] and the lesions of white matter (WM) in the pons, the frontal, the temporal and the parietal cortices, the corpus callosum, projections to and from the cerebellum, and within the limbic system [17], [18]. All those studies suggested multiple regional brain changes could have been occurred in chronic hypoxia-exposed HA residents.

The prefrontal cortex, the insular cortex, and the cingulate cortex have been proved involved in cardiovascular control [19]–[21], and among those brain areas, anterior insular and anterior cingulate cortices play an important role in the unpleasantness of dyspnea [22], which often occurred during the adaptation to HA hypoxia. Recently, Paulus et al. [23] proposed the hypotheses that the anterior insular and cingulate cortices should be needed to process perturbation of the homeostatic balance in extreme environments. Aerobic capacity was strongly correlated with the right anterior insular GM density [24] and decrease in aerobic capacity has been well known in sea-level (SL) residents who were acclimatized to chronic HA hypoxia during the developmental period [25], [26]. Thus, a decreased GM in anterior insula at residents acclimatized to HA was suggested. Based on the above data, we expected that, in order to adapt changed peripheral physiology at HA environment, the modifications in brain may include these areas.

In this study, to investigate structural modifications in brain, residents in the Qinghai-Tibetan Plateau were examined using magnetic resonance imaging (MRI). Voxel-based morphometry (VBM) provides a quantitative and comprehensive assessment of anatomical differences throughout the brain [27]. It had been widely applied in numerous clinical and neuroscience researches. In the present study, we used VBM to reveal possible changes in GM volume. Diffusion tensor imaging (DTI) produces in vivo images of biological tissue. The anisotropy of the fibrous microstructure reflected from restricted water diffusion is measured with fractional anisotropy (FA) value. FA can reflect neural axons of the WM in the brain and used as a measure for WM integrity [28]. FA values have successful clinical application [29]–[31]. Current DTI data analyses of FA values include region-of-interest (ROI) analysis and voxel-wise analysis. We first used conventional regional analyses to characterize the pattern of anatomic alterations of HA effects by examining multiple, separate ROIs that spanned all brain structures. This anatomic structure based ROI analysis of FA values has been successful used to study clinical diseases [32]–[34]. Tract-Based Spatial Statistics (TBSS) is a new voxel-wise method proposed recently. It alleviates the alignment-related problems by applying both linear and nonlinear alignment to data and additionally projecting the FA values of individual subjects in given spatial locations to the thinned common “FA-skeleton” of major WM structures, thus improving sensitivity, objectivity, and interpretability of analysis of multi-subject DTI data [35], [36]. Moreover, diffusion tensor eigenvalues (longitudinal and radial diffusivities) were also included in the analysis since they can help interpret FA changes in WM tracts by providing information regarding likely alterations in the proportion of longitudinally vs. obliquely aligned myelinated fibers [37]. TBSS has also been successful used to study clinical diseases [37]. In the present study, using TBSS, we measured FA and longitudinal and radial diffusivities to examine alterations in alignment of myelinated fibers in WM tracts in the whole brain.

The influence of hypoxic stress, and the organism's response to it, are greater during growth than during adulthood. The differences between the highland and lowland natives in their physiological performance and morphology are mostly due to adaptations acquired during the developmental period [38]. The study on the Bolivians of foreign ancestry acclimatized to high altitude since birth or during growth attained suggested that developmental acclimatization is important in the attainment of normal physiological functions at HA [39]. Therefore, HA young adult residents (range 17–22 yr) born and raised at HA were studied in the present study. All of their ancestors migrated from SL.

## Results

### Physiological characteristics

There were no significant differences in hemoglobin levels, circulating red blood cell count, blood pressure, and pulse rate between HA residents and SL controls. Body height of both males and females in HA residents were significantly larger than that of their SL controls (*p* = 0.006; *p* = 0.015, respectively). HA females had a higher diastolic pressure (*p* = 0.017) compared with SL females. No significant differences in pulmonary function and hematological measurements were found between HA residents and SL subjects (Table 1).

**Table 1:** Physiological Characteristics.

	HA		SL	
	Males	Females	Males	Females
**Body Height (cm)**	176.7±4.6**	163.3±5.4#	171.2±5.5	159.1±5.4
**Body Weight (kg)**	59.3±6.0	50.5±5.3	57.0±5.9	50.2±4.6
**Hematological measurements**				
HGB (g/L)	145.60±9.45	136.60±7.27	149.0±3.1	132±2.1
RBC (×10^12^)	5.02±0.61	4.45±0.28	5.08±0.7	4.33±0.4
**Blood pressure** (Kpa)				
Systolic pressure	119.8±10.3	110.3±6.6	115.3±11.4	108.5±7.4
Diastolic pressure	75.2±5.2	77.0±9.6#	76.6±9.6	67.3±4.7
**Pulse Rate** (times/min)	70.6±11.6	76.9±6.8	77.4±6.6	74.9±4.8
**Pulmonary Function testing**				
VC (liters)	2.97±1.64	3.19±0.51	3.72±0.30	2.78±0.34
IRV (liters)	1.12±0.69	1.20±0.38	2.06±0.66	1.09±0.34
TV (liters)	0.86±0.41	0.73±0.19	0.68±0.38	0.64±0.30
IC (liters)	1.59±1.04	1.88±0.38	2.25±0.50	1.56±0.34
MVV (real/estimated %)	143.64±29.92	104.03±28.66	141.29±29.95	97.84±14.09
RR (times/min)	19.61±7.41	19.58±3.31	16.58±5.12	18.12±6.45
FVC (real/estimated %)	95.23±31.47	96.64±11.13	92.86±5.72	100.21±14.88
FEV1 (real/estimated %)	101.07±26.96	104.10±7.29	92.00±8.81	81.63±36.21
%FEV1(real/estimated %)	110.68±7.67	117.26±8.43	101.95±7.16	89.63±39.81
MMEF(real/estimated%)	87.98±23.42	89.95±14.87	81.36±25.68	66.09±39.09
PEFR (real/estimated %)	94.13±40.03	93.52±9.97	87.64±17.68	66.66±35.11

VC, vital capacity; TV, tide volume; IRV, inspiratory reserve volume; IC, inspiratory capacity; MVV, maximum voluntary ventilation; RR, respirotary rate; FVC, forced vital capacity; FEV1, forced expired volume in one second; MMEF, mean mid-expiratory flow; PEFR, peak expiratory flow rate. Data are Mean (SD).

**p<0.01 vs. SL males;

# *p*<0.05 vs. SL females.

### Average volume of GM, WM and cerebrospinal fluid (CSF)

No subject from either group showed visible abnormalities on T1-weighted structural images. There were also no significant differences in average volumes of the whole brain, the GM or the WM between the two groups; but HA residents showed a significant increase in CSF total volume (*t* = 2.302, *p* = 0.025) (Table 2).

**Table 2:** Average volume (mm^3^) of GM, WM and CSF in HA and SL groups (mean ± SD).

	HA	SL	*t*	*p*
GM	691.33±75.24	704.87±78.89	0.658	0.513
WM	393.66±44.97	391.66±30.36	0.197	0.845
CSF	332.55±69.82	292.85±58.73	2.302	0.025

GM, gray matter; WM, white matter; CSF, cerebrospinal fluid.

### GM

**Figure 1:**
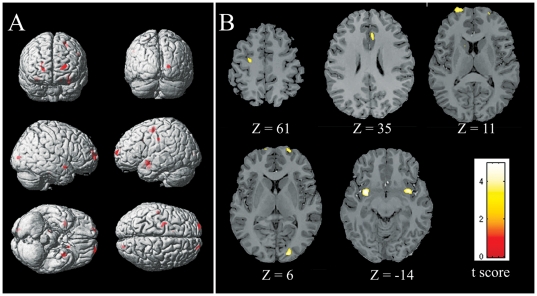
Gray matter volume decrease in HA residents vs. SL controls. (A) A statistical parametric map for gray matter reduced in HA residents compared with SL controls (B) Axial slice series depicting regions showing reduced gray matter volume in HA residents overlaid on Talairach template. Display threshold was set at |*t*|>2.70, *p*<0.01 (FWE corrected). Detailed coordinate and cluster size information of significant regions were shown in Table 3.

VBM analysis showed that HA residents had decreased GM volume compared with SL controls in the bilateral anterior insula, the right anterior cingulate cortex, the bilateral prefrontal cortex, the left precentral cortex, and the right lingual cortex. (two-sample *t*-test, |*t*|>2.70, *p*<0.01, FEW corrected) (Figure 1). Coordinate information is shown in Table 3.

**Table 3:** Regional information of decreased gray matter volume in HA subjects compared with SL controls.

Area	Sides	Cluster size(mm^3^)	BrodmannArea	MNI coordinates (peak)			t
				x	y	z	
Precentral cortex	L	2006	6	22	16	63	−3.54
Insula	L	1556	13	36	−2	−5	−4.95
	R	811	13	−36	−5	−3	−4.34
Lingual cortex	R	579	17	−19	90	5	−4.04
Cingulate cortex	R	532	32	10	−9	42	−3.33
Prefrontal cortex	R	502	10	−24	−59	20	−3.52
	L	485	9	23	−60	26	−3.77

Coordinate and t value are from the voxel with the peak t value. Negative *t* value means decrease in HA subjects. (|*t*|>2.70, *p*<0.01, FWE corrected).

### FA

#### ROI analysis

ROI analysis revealed significant increase of FA values in both the right and left anterior limb of internal capsule (ALIC) and significant decrease at the right posterior cingulum in HA residents compared with SL controls. No significant differences were detected in other areas. Mean FA values of each ROI for HA residents and SL controls are shown in Table 4 (two-sample *t*-test, *p*<0.05).

**Table 4:** Detailed regional FA value (mean ± SD) changes in HA subjects compared with SL controls.

ROIs	Sides	HA	SL	*t*	*p*
CC					
Rostrum		0.78±0.05	0.77±0.02	0.646	0.522
Genu		0.80±0.04	0.79±0.01	1.385	0.174
Rostral body		0.76±0.03	0.75±0.02	0.513	0.611
Anterior midbody		0.75±0.03	0.75±0.03	0.283	0.779
Posterior midbody		0.76±0.04	0.77±0.03	0.442	0.661
Isthmus		0.74±0.05	0.73±0.04	0.294	0.770
Splenium		0.79±0.03	0.79±0.02	0.248	0.782
PFWM	R	0.51±0.03	0.52±0.04	0.955	0.345
	L	0.54±0.04	0.55±0.02	0.925	0.361
TLWM	R	0.47±0.02	0.46±0.03	0.498	0.622
	L	0.51±0.03	0.52±0.02	0.413	0.631
OLWM	R	0.47±0.05	0.48±0.05	0.496	0.623
	L	0.48±0.04	0.48±0.04	0.035	0.972
IC					
Anterior limb	R	0.65±0.04	0.60±0.04	2.782	0.008
	L	0.64±0.04	0.61±0.03	2.874	0.007
Genu	R	0.68±0.03	0.67±0.04	1.674	0.102
	L	0.68±0.03	0.67±0.04	0.737	0.466
Posterior limb	R	0.70±0.03	0.69±0.02	0.346	0.731
	L	0.72±0.03	0.70±0.02	1.394	0.171
Cingulum					
Anterior part	R	0.55±0.06	0.53±0.05	1.070	0.291
	L	0.49±0.04	0.49±0.05	0.492	0.626
Middle part	R	0.56±0.04	0.57±0.03	0.564	0.576
	L	0.56±0.05	0.56±0.03	0.130	0.897
Posterior part	R	0.52±0.05	0.56±0.04	2.934	0.022
	L	0.59±0.05	0.59±0.04	0.248	0.806
EC					
Anterior part	R	0.56±0.03	0.56±0.03	0.130	0.897
	L	0.56±0.04	0.57±0.03	0.529	0.600
Middle part	R	0.49±0.04	0.49±0.05	0.492	0.626
	L	0.55±0.06	0.53±0.05	1.045	0.302
Posterior part	R	0.52±0.03	0.51±0.04	0.699	0.489
	L	0.56±0.05	0.54±0.04	0.857	0.397

CC, corpus callosum; EC, external capsule; IC, internal capsule; OLWM, occipital lobe white matter; PFWM, prefrontal white matter; TLWM, Temporal lobe white matter.

#### Whole brain voxel-wise statistic analysis

**Figure 2:**
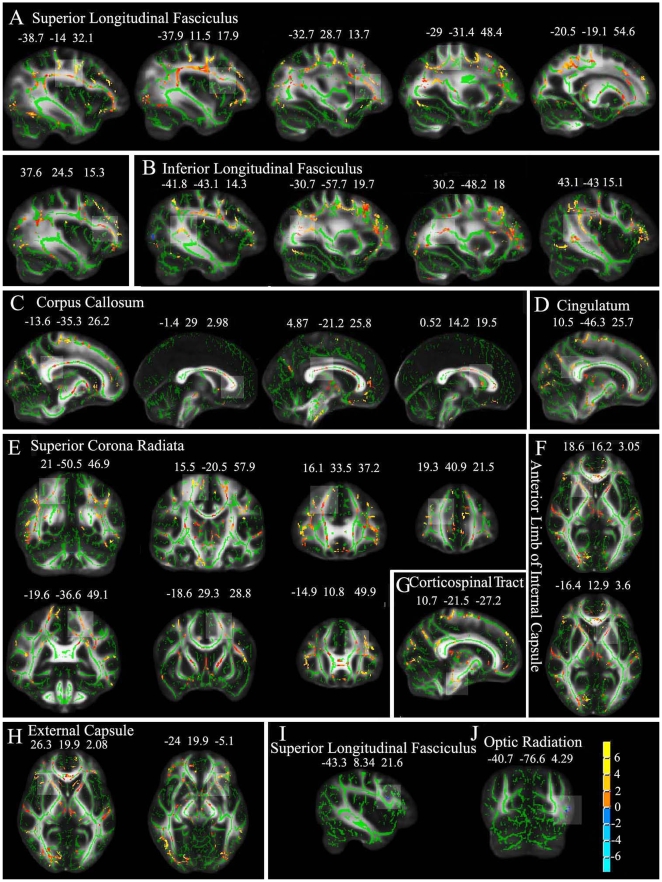
Statistical maps of group comparison of FA values on a voxel-wise basis (results of TBSS). The group's mean FA skeleton (green) was overlaid on the mean FA images. The threshold of mean FA skeleton was set at 0.2. (A–H) show significantly higher FA value (red to yellow) and (I and J) show significantly lower FA value (blue) in HA residents than SL controls at p<0.05. Detailed coordinate and cluster size information of significant regions (higher FA: clusters >100 voxels; lower FA: clusters >8 voxels) were shown in Table 5.

Whole brain voxel-wise statistic analysis showed HA residents had significantly higher FA in a broad range of brain areas compared with SL controls (*p*<0.05, uncorrected) (Figure 2, Table 5). The significant regions (clusters size>100 voxels) included the right and left superior longitudinal fasciculus (SLF), the right and the left inferior longitudinal fasciculus (ILF), the corpus callosum (genu and body), the right and left superior corona radiata (SCR), the right and left ALIC, the right and left anterior external capsule, the right posterior cingulum and the right corticospinal tract. Conversely, compared with SL subjects, there were some regions with lower FA values in HA residents at the left optic radiation and left SLF (the inferior frontal lobe, the pars opercularis, the BA 44) (clusters size >8 voxels, Table 5).

**Table 5:** Main regions showing greater (clusters >100 voxels) and reduced (clusters >8 voxels) FA in HA subjects and SL controls.

MNI coordinates			Voxels(mm^3^)	White matter tract	Corresponding cortical area	FA Mean (SD)		Diffusivity Values (×10^−3^ mm^2^/s)			
(peak)						HA	SL	Longitudinal		Radial	
x	y	z						HA	SL	HA	SL
**Increased FA: HA vs. SL**											
−38.7	−14	32.1	1514	SLF-L	Frontal precentral gyrus	0.501(0.066)	0.478(0.064)	1.101(0.085)	1.049(0.077)	0.741(0.089)	0.771(0.109)*
−37.9	11.5	17.9	212	SLF-L	Frontal opercular cortex	0.511(0.049)	0.477(0.059)	0.969(0.019)	1.121(0.094)	0.808(0.159)	0.839(0.171)*
−32.7	28.7	13.7	191	SLF-L	Frontal lobe, BA 11	0.457(0.027)	0.422(0.035)	0.992(0.056)	1.104(0.010)	0.704(0.081)	0.745(0.097)
−29	−31.4	48.4	108	SLF-L	Frontal Sub-Gyral lobe	0.516(0.043)	0.483(0.045)	1.038(0.054)	1.221(0.084)	0.771(0.081)	0.852(0.134)
−20.5	−19.1	54.6	182	SLF-L	Parietal postcentral gyrus	0.586(0.049)	0.570(0.048)	1.100(0.123)	1.159(0.082)	0.683(0.089)	0.760(0.155)*
37.6	24.5	15.3	400	SLF-R	Inferior frontal pars triangularis	0.457(0.062)	0.423(0.053)	1.002(0.009)	1.004(0.098)	0.854(0.159)	0.916(0.164)*
−41.8	−43.1	14.3	395	ILF-L	Posterior thalamus	0.562(0.077)	0.529(0.082)	1.092(0.042)	1.059(0.144)	0.742(0.044)	0.716(0.062)
−30.7	−57.7	19.7	322	ILF-L	Temporal pianum temporle lobe	0.566(0.085)	0.530(0.080)	1.334(0.196)	1.210(0.074)	0.755(0.050)	0.757(0.065)
43.1	−43	15.1	534	ILF-R	Temporal supramarginal gyrus	0.525(0.055)	0.496(0.056)	1.115(0.087)	1.260(0.069)	0.741(0.052)	0.773(0.058)*
30.2	−48.2	18	100	ILF-R	Temporal lobe	0.578(0.035)	0.547(0.043)	1.387(0.131)	1.434(0.105)	0.679(0.070)	0.676(0.033)
−1.4	29	2.98	334	CC	Genu	0.798(0.044)	0.775(0.054)	1.447(0.192)	1.424(0.115)	0.771(0.143)	0.822(0.146)*
0.52	14.2	19.5	160	CC	Anterior body	0.681(0.066)	0.648(0.070)	1.514(0.144)	1.522(0.095)	0.871(0.161)	0.908(0.136) *
4.87	−21.2	25.8	129	CC	Posterior body	0.650(0.096)	0.612(0.101)	2.248(0.253)	1.884(0.176)	0.778(0.190)	0.970(0.266) *
−13.6	−35.3	26.2	150	CC	Posterior body	0.774(0.041)	0.753(0.044)	2.290(0.411)	1.971(0.065)	0.682(0.175)	0.982(0.282)**
19.3	40.9	21.5	130	SCR-R	Frontal pole, BA 9	0.504(0.026)	0.474(0.024)	1.245(0.061)	1.140(0.079)	0.906(0.133)	0.906(0.139)
16.1	33.5	37.2	142	SCR-R	Frontal pole, BA 8	0.499(0.044)	0.476(0.043)	1.071(0.015)	1.162(0.077)	0.884(0.070)	0.928(0.104)*
15.5	−20.5	57.9	560	SCR-R	Frontal precentral gyrus	0.548(0.078)	0.530(0.073)	1.161(0.076)	1.169(0.103)	0.812(0.122)	0.909(0.179)*
21	−50.5	46.9	146	SCR-R	Parietal precuncus lobe	0.551(0.025)	0.535(0.035)	1.179(0.097)	1.148(0.075)	0.850(0.065)	0.964(0.110)*
−18.6	29.3	28.8	186	SCR-L	Paracingulate gyrus	0.539(0.041)	0.507(0.049)	1.093(0.021)	1.158(0.069)	0.705(0.035)	0.726(0.062)*
−14.9	10.8	49.9	124	SCR-L	Paracingulate gyrus	0.566(0.067)	0.528(0.082)	1.066(0.141)	0.502(0.069)	0.859(0.121)	0.899(0.173)*
−19.6	−36.6	49.1	1439	SCR-L	Postcentral gyrus	0.537(0.057)	0.506(0.056)	1.165(0.094)	1.175(0.092)	0.747(0.096)	0.814(0.139)*
18.6	16.2	3.05	215	ALIC-R	ALIC	0.572(0.077)	0.524(0.070)	1.218(0.072)	1.086(0.086)	0.577(0.076)	0.619(0.119)*
−16.4	12.9	3.6	203	ALIC-L	ALIC	0.607(0.072)	0.567(0.069)	1.054(0.068)	1.158(0.088)	0.600(0.024)	0.598(0.039)
26.3	19.9	2.08	115	EC-R	Anterior insula	0.531(0.057)	0.506(0.055)	1.023(0.036)	1.085(0.076)	0.616(0.032)	0.633(0.036)*
−24	19.9	−5.1	112	EC-L	Anterior insula	0.564(0.063)	0.518(0.044)	1.108(0.087)	1.024(0.083)	0.592(0.074)	0.608(0.069)*
10.5	−46.3	25.7	101	CG-R	Posterior cingulum	0.534(0.057)	0.508(0.061)	1.192(0.136)	1.170(0.061)	0.597(0.042)	0.582(0.038)
10.7	−21.5	−27.2	157	CT-R	Ventral pons	0.644(0.080)	0.606(0.077)	1.906(0.176)	2.068 0.014)	1.485(0.270)	1.156(0.113)
**Decreased FA: HA vs. SL**											
−40.7	−76.6	4.29	14	OR-L	Lateral occipital cortex, V5	0.187(0.006)	0.295(0.027)	0.872(0.086)	0.641(0.199)	0.736(0.098)	0.698(0.034)
−43.3	8.34	21.6	9	SLF-L	Inferior frontal lobe, BA 44	0.199(0.022)	0.238(0.006)	1.00(0.177)	0.751(0.211)	0.842(0.050)	0.836(0.051)

Cluster size (*p*<0.05, uncorrected) and the location of its peak value in the cluster. SLF-L, Left superior longitudinal fasciculus; SLF-R, Right superior longitudinal fasciculus; ILF-L, Left inferior longitudinal fasciculus; ILF-R, Right inferior longitudinal fasciculus; CC, Corpus callosum; SCR-L, Left superior corona radiata; SCR-R, Right superior corona radiata; ALIC-L, Left anterior limb of internal capsule; ALIC-R, Right anterior limb of internal capsule; EC-L, Left external capsule; EC-R, Right external capsule; CG-R, Right cingulatum; CT-R, Right corticospinal tract; OR-L, Left optic radiation; BA, Brodmann area.

*p<0.05;

**p<0.01.

#### Radial diffusivity

Higher FA (clusters >100 voxels) in the regions of the right and left SLF (frontal precentral cortex, frontal opercular cortex, parietal postcentral gyrus, inferior frontal pars triangularis), the right ILF (temporal supramarginal gyrus), the right and left SCR (frontal pole, BA 8, frontal precentral gyrus, parietal precuncus lobe, paracingulate gyrus, and postcentral gyrus), the right ALIC, the right and left external capsule (the anterior insula), and the corpus callosum (genu and body) were associated with decreased radial diffusivity in HA residents compared with SL controls. No significant differences were detected in the higher FA regions of the left SLF (the frontal lobe, BA 11 and the frontal Sub-Gyral lobe), the right and left ILF (the posterior thalamus, the temporal lobe and the temporal pianum temporle lobe), the right SCR (the frontal pole, BA 9), the left ALIC, the right posterior cingulatum, and the right corticospinal tract between the HA and SL groups. Lower FA values (clusters size >8 voxels) in the both left optic radiation (V5) and left SLF (inferior frontal lobe, BA 44) were associated with no changed radial diffusivity in HA subjects compared with SL controls. (Table 5)

#### Longitudinal diffusivity

All the regions have no significant changes in the longitudinal diffusivity between HA and SL groups (Table 5).

## Discussion

Our present study first revealed that HA acclimatization was associated with brain structural modifications, which included the loss of regional cortical GM volume and WM structures. As we anticipated, the changed GMs were mainly confined in the prefrontal cortex, the anterior insular cortex, the anterior cingulate cortex, and the lingual cortex. Importantly, we found significant changes in anisotropy and diffusivity that reflect widespread alterations in fiber pathways. No significant differences were found in the average volume of GM, WM, and the whole brain of HA residents compared to SL controls, indicating that no global brain changes occurred during the acclimatization to HA. Higher CSF in HA residents may be a sign of a larger ventricle space in HA than in SL brain. Since our subjects had lived at SL for over one year, the present study suggested that the structural changes persisted even after HA residents had relocated at normoxia environment for a long time.

### The implications for the loss of GM in the anterior insula, the anterior cingulate cortex, and the prefrontal cortex

Our present findings confirmed the hypotheses proposed by Paulus et al. [23] that the anterior insular and cingulate cortices should be needed to process perturbation of the homeostatic balance in extreme environments and agreed with the results of MRI studies on hypoxic OSA patients who showed a loss of anterior cingulate GM [13], [18].

The insular cortex is connected with the hypothalamic, the midbrain, the pontine, and the medullary brain regions that are involved in cardiovascular control and receives visceral sensory information arising from baroreceptors and chemoreceptors within the cardiovascular system [19]. The information is then relayed to the anterior cingulate cortex [23]. Electrical cortical stimulation and human neuroimaging studies have demonstrated that the activation of the anterior insula, the dorsal anterior cingulate cortex and the prefrontal cortex, modulated sympathetic nerve activity, heart rate, and blood pressure [21], [24], [40]–[42]. Dyspnea, often occurred during the adaptation to HA, activated the insular cortex, which had been shown in the inspiratory loading and volitional breathing tasks [2], [22], [43], [44]. Therefore, elevations in the sympathoadrenal system in the SL natives temporarily exposed to HA may be due to the activity of the anterior insula and its connected brain areas. HA hypoxia was also reported to have induced activation of the carotid baroreceptors, but it was not sufficient to completely counteract the catecholamine-induced increase in blood pressure and peripheral vasoconstriction [45]. However, after some years of residence the blood pressure tends to gradually decline, even falling below that observed at SL [46]. The decreased GM volume in the anterior cingulate and the insular cortices that found in the present study might be responsible for the decreased blood pressure. As the lesion to the insula has been proved to disrupt the representation of internal states that underpinning motivation [47], reduce in the anterior insular GM may be a reason why HA residents had a blunted hypoxic ventilatory response [48].

The HA residents have not shown significant differences with their SL peers in the peripheral physiology after they had relocated SL for over one year, which suggested pulmonary and cardiovascular functions had readapted to SL after acclimatization to HA [2], [3]. However, possibly due to failure in neuronal regenerate in SL normoxia, the loss of brain GM persisted. This may explain why HA residents persisted blunting of the ventilatory response to hypoxia [49] and had the lesser degree of sympathetic activation [45] despite long-term acclimatization at SL. One shortcoming of the present study was that we did not test hypoxic ventilatory response.

The loss of GM in the anterior insula, the prefrontal cortex, and the anterior cingulate cortex found in the present study may clarify the mechanisms underlining the decreased aerobic capacity [24]–[26] and decreased appetite [50] and may suggest changed cognitive functions [51]–[53] in HA acclimatized immigrants.

### The implications for the changes in WM

In the present study, although anatomic structure based ROI analysis only detected changed FA confined to a few areas, changed FA values were found in a broad areas of WM using TBSS analysis. One of the most interesting findings of our study is the changes in FA values were symmetric in most regions between the left and right hemispheres in HA residents except the superior longitudinal fasciculus. The symmetric FA increase in both hemispheres implies the equal impacts of HA on the left and right sides of the brains. The discrepant findings between ROI and TBSS analyses may be due to that ROI analysis is difficult to objectively and reproducibly place ROIs on small or thin tracts on the images of individual subjects, when the slice orientation and anatomical details may show variation between individuals and the boundaries of the WM tracts are not easily identified. Moreover, the FA value obtained from ROI analysis is largely affected by location and size of the ROIs; both being limiting factors in accuracy of ROI method since the ROI selection is often conducted without a prior knowledge about the exact location [54]. However, both two analysis methods consistently showed significantly higher FA in the bilateral ALIC.

Although the voxel-based analysis provides the ability to map the FA changes within the whole brain at once without a priori define ROIs it has been shown that the interpretability of the results from voxel-based DTI may sometimes be questionable [55], [56]. The TBSS method used here overcomes the problems in the voxel-based DTI analysis in regards of alignment and spatial smoothing, and thus seems to be a far more accurate voxelwise analysis method when comparing multi-subject diffusion data [35]. Greater FA may reflect greater myelination of WM fibers, increased number of myelinated fibers, smaller axonal diameter, or reduced neural branches within MRI voxel [37], [57], [58]. Whereas reduced FA was associated with local cerebral edema, cerebrospinal fluid, compromised myelin structure, changes in axonal morphologic structure, and altered interaxonal spacing of fiber bundles [57], . In our study, further analysis found that most of those regions were associated with reduced radial diffusivity coinciding with no change of longitudinal diffusivity. Radial diffusivity was positively correlated with the mean axon diameter while the longitudinal diffusivity was negatively correlated with it [63]. Greater FA values associated with reduced radial diffusivity coinciding with no change of longitudinal diffusivity can result from increased myelin thickness, smaller axonal diameter or extracellular space [64].

The ALIC contains the anterior thalamic peduncle, which connects the dorsomedial and anterior thalamic nuclei with the prefrontal cortex and the cingulate cortex. From a functional neuroanatomic perspective, ALIC is involved in the medial limbic circuit (composed of the hippocampal formation, the mammillary bodies, the anterior thalamic nuclei, and the cingulate gyrus) and the basolateral limbic circuit (interconnecting the orbitofrontal cortex, the dorsomedial thalamic nucleus, the amygdala, and the anterior temporal cortex) [65]. The increased FA values with reduced radial diffusivity and no change of longitudinal diffusivity in ALIC described in our study indicated that HA acclimatization was involved with improvement of the fronto-thalamic or cingulate-thalamic structural connectivity, which may result in higher functional connectivity between the cortical cortex and subcortical regions partially linked by these circuits.

The changed FA in the posterior cingulate fiber tract may alter communication between components of the Papez circuit in HA residents [66]. The body of the corpus callosum contains fibres important for connecting motor and sensory cortices. The radiating bundle of corona radiata fibres generates ascending paths to the motor cortices and descending paths such as the corticospinal tract to spinal motor neurons. Therefore, increases of FA at those locations may be linked to improvement of motor skills in acclimatization to HA.

### The mechanisms that involved in brain structural modulations

The structural changes in the brain of HA residents may be related to the decrease in glucose metabolism [7] and/or impaired cerebral autoregulation [8], [9]. Moreover, GM loss mostly occurred in new cortex in HA residents found in the present study may be due to the unevenly distributed cerebral blood flow and cerebral blood volume during baseline conditions. Previous study revealed that phylogenetically older regions of the brain, which receives greater than average increases in cerebral blood flow, showed larger vascular responses to hypoxia than evolutionary younger regions that generally received below average increases [67].

### What determined the brain structural modifications: gene or developmental environment?

The brain structural modifications found in our subjects who ancestors migrated from SL and have resident at HA at least two generations may occur in genetically adapted HA natives, such as Tibetan, and in adolescents who immigrated to HA with parent after their birth. A recently study of Hogan et al. [68] may support our hypothesis. Hogan et al.([68] tested a reduced psychomotor speed in children (6–10 years) and adolescents (13–16 years) of mixed-ethnic background (including Native American, European or African ancestry whose ancestor immigrate to HA during different periods) who were born and raised at HA, they found the reduced psychomotor speed was well correlated with the reduced cerebral metabolism and blood flow. For the proportion of European, Native American and African genetic admixture was comparable across altitude groups, therefore, chronic hypoxic exposure rather than genetic inheritance appears to affect the neurocognitive development at HA. However, Brutsaert et al. [69] found that pulmonary function (forced vital capacity and forced expiratory volume) measures were larger in HA natives compared with low altitude natives born and raised at HA, suggesting a genetic effect. In contrast, forced vital capacity and forced expiratory volume were similar in HA natives and SL natives at low altitude, suggesting that the genetic potential for larger lung volumes at HA depended upon developmental exposure to HA. In summary, results from those studies emphasized the importance of developmental adaptation to HA.

As our present findings in white matter change regions are in agreement with most of regions showing FA increases during adolescence [70], we speculate that higher FA with decreased radial diffusivity coinciding with no change of longitudinal diffusivity is a developmental modification in fiber microstructure in acclimatization to HA. Recently, through longitudinal studies, Bava et al. [70] have documented linear increases in FA with decreases in radial diffusivity across typical adolescent development continuing through the second decade of life, showing FA increases in the bilateral SLF, SCR, thalamic radiations, posterior internal capsule, corticospinal tract, arcuate fasciculus, superior and mid-temporal white matter, inferior parietal white matter, and the corpus callosum. Future study should be done to explore how the brain acclimatized to HA in residents who immigrated from SL to HA at adult and have been resided at HA for many years.

In summary, we demonstrated that HA acclimatization was associated with regional brain structural modifications, which mainly related to cardiovascular or respiratory regulations. These regional brain changes may underlie the physiological functions of HA residents at high altitude and indicate some cognition and motor skill deficits or enhancement. These changes persisted even after HA inhabitants relocated to a SL residence. Future study is needed to explore the functional modifications of the brain in acclimatization to HA and structural brain modulations in relation to the behaviors and physiological functions of HA residents.

## Materials and Methods

### Subjects

Subjects in the current study consisted of 28 HA immigrant residents (mean age 20.4 yr; range 17–22 yr) born and raised at the altitude of 2616–4200 m in Qinghai-Tibetan Plateau for at least 17 years and currently attended college at Chendu (<400 m), and their ancestors migrated from SL and have resident at HA at least two generations (Table 6). The 28 matched control subjects (mean age 20.9 yr; range 17–23 yr) were their schoolmates, all of whom were lowlanders, born and living at sea level below 400 m and without any prior exposure to high altitude. HA and SL students did not differ in IQ or college enrollment scores and all of them were selected from Han populations to avoid possible racial differences. Physiological and MRI studies were examined at Chendu. Subjects were excluded if they had: (1) chronic mountain sickness, (2) a documented neurological disorder, or (3) a past history of head injury with loss of consciousness. Procedures were fully explained, and all subjects provided written informed consent before participating in the study. The experimental protocol was approved by the Research Ethics Review Board of the Institute of Psychology, Chinese Academy of Sciences.

**Table 6:** Demographic information of HA subjects and SL controls (based on self-reported).

	HA	SL
Number of subjects	28 (male:12, female:16)	28 (male:12, female:16)
Ages (mean ± SD) (yrs)	20.4±1.4	20.9±1.5
Altitudes of residence (mean ± SD) (m)	2982.8±478.7	<400
Generations of residence at HA	2 (28%), 3 (43%),>3 (29%)	-
Time of relocation at SL (yrs)	1 (18%), 2 (32%), 3 (50%)	-
Education level (mean ± SD) (yrs)	13.3±0.8	13.4±0.6
Education of parents (mean ± SD) (yrs)	8.8±3.4	9.2±1.8

### Physical and physiological assessments

Before MRI scanning, subjects underwent physical and physiological examinations, including body weight, hematological measurements, blood pressure, pulse rate, and pulmonary function. Hematological measurements were tested using a hematology analyzer (Sysmex XE-2100, TOA Medical Electronics, Kobe, Japan). Pulmonary function was tested using a pulmonary function testing device (Master Screen Body, JAEGER, German).

### MRI data acquisition

Structural images were acquired on a GE 3.0 T Signa Excite Gemse MRI system (GE Medical, Milwaukee, WI, USA) at Huaxi Magnetic Resonance Research Center (West China Hospital, Chengdu, China).

A 3D structural MRI was acquired from each subject using a T1-weighted MPRAGE sequence (TR/TE = 8.5 ms/3.4 ms, TI = 400 ms, FOV = 280×280 mm^2^, in-plane resolution = 0.547×1.094 mm^2^, flip angle = 12°), yielding 156 contiguous axial slices (1 mm thick) covering the whole brain. A DTI pulse sequence with single shot diffusion-weighted echo planar imaging (TR/TE = 10000/70.8 ms, FOV = 240×240 mm^2^, in-plane resolution = 1.875×1.875 mm^2^) was applied sequentially in 16 different directions. We acquired 42 contiguous 3-mm thick slices covering the whole brain.

### VBM analysis of 3D T1 images

The 3D T1 images were used for GM volume analysis using VBM implemented in SPM2 toolbox (Wellcome Department of Imaging Neuroscience, London). For each section of GM, WM and cerebral spinal fluid compartments, we constructed an anatomical template map as well as a probability map specific to the current study based on the 3D images from all the subjects. Using these study-specific templates, the 3D images for each individual were spatially normalized to Talairach space [71] and segmented, and then were smoothed using a Gaussian kernel of 10 mm full-width at half-maximum (FWHM). Random-effect two-sample *t*-tests were performed to examine between-group differences. The statistical parametric map was generated with threshold at |*t*|>2.70, *p*<0.01 (FWE corrected).

### Whole brain voxel-wise statistic analysis and ROI analyses of DTI

#### ROI analysis

**Figure 3:**
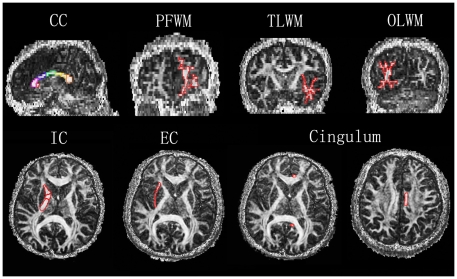
Examples of ROI demarcation on FA images. CC: Corpus callosum; PFWM: Prefrontal white matter; TLWM: Temporal lobe white matter; OLWM: Occipital lobe white matter; IC: Internal capsule; EC: External capsule. The ROIs were defined as Table 7.

All analysis were conducted with the DTI Studio software version 2.40 provided by Johns Hopkins University [72] (https://www.dtistudio.org/) on the FA image of each participant. The appropriate slice on which the ROI was identified was chosen by using the anatomical landmarks from the FA maps. The ROIs were then drawn manually by an experienced operator blind to subjects' status, using standardized guidelines based on location and size. ROIs were placed in a total of 10 areas. The ROIs were depicted in Figure 3 and defined as Table 7. Data were further analyzed using SPSS. ANOVA statistic identified the differences between the HA and SL groups. Data were presented as mean±SD. Statistical significance was set at *p*<0.05.

**Table 7:** Regional definitions for FA values analyses (Depicted in Figure 3).

Corpus callosum (CC)	A semiautomated subregional division of the CC, as described by Witelson [74] was implemented in which the CC was divided into seven segments: the rostrum, genu, rostral midbody, anterior midbody, posterior midbody, isthmus, and splenium.
Prefrontal white matter (PFWM)	Coronal slice: The most anterior slice on which the PFWM was measured was at the rostral point of the cingulate sulcus and the most posterior slice was the slice immediately anterior to the genu of the corpus callosum [32].
Temporal lobe white matter (TLWM)	Coronal slices: Slice beginning at the mammillary bodie and ending at the posterior commissure [32].
Occipital lobe white matter (OLWM)	Coronal slices: The most anterior slice on which the OLWM was measured was at the posterior point of the cingulate sulcus.
Internal capsule (IC)	Axial slice: The anterior limb was sampled between the pallidum and head of the caudate nucleus; The posterior limb and the genu of the internal capsule was sampled between the head of the caudate nucleus and the pallidum and by the pallidum and the thalamus.
Cingulum	Axial slice: The anterior and the posterior portion of the cinglum on the same axial slices as the body of the fornix; The middle portion of the cingulum identified on an axial slice just superior to the body of corpus callosum.
External capsule (EC)	Axial slice: The anterior, middle and the posterior portion of the external capsule were identified on the same axial slices that identified internal capsule.

#### Whole brain voxel-wise statistic analysis

We used DCM2MII to convert diffusion tensor images from the proprietary scanner format to the NIFTI format. Then images were processed using FSL 4.1.5 software package (http://www.fmrib.ox.ac.uk/fsl/). Images were realigned to the b-value (b0) image to remove eddy current distortions and motion artifacts using FDT (FMRIB's diffusion toolbox) [73]. Brain mask was created from the first b0 image using BET (Brain Extraction Tool). After those processes images were calculated with the FDT for FA and longitudinal diffusivity and radial diffusivity maps. The analysis of FA images was performed using the TBSS package in FSL [35], [36]. TBSS processing includes the following steps: (1) Align the FA images of all subjects to a template which was arbitrarily selected from those FA images by nonlinear registrations; (2) Transform all the aligned FA images into 1×1×1 mm^3^ MNI152 space by affine registrations; (3) Create the mean FA image and filter to retain only the center of the WM tracts so as to create the mean FA skeleton; (4) Project individual subjects' FA was put onto the skeleton. (5) Following these steps, data were fed into voxel-wise cross-subject statistical analyses with the following group comparisons: HA vs. SL and SL vs. HA. In all cases, the null distribution was built up over 5000 permutations, and significance was tested at *p*<0.05 levels, uncorrected for multiple comparisons. We determined the anatomic localization of each cluster by means of the FSL atlas tool, which incorporates several anatomic templates, including the Talairach atlas, MNI structural atlas, Julich histological atlas, Harvard-Oxford cortical and subcortical structural atlases, and the Johns Hopkins University DTI-based WM atlases.

Longitudinal (principal diffusion direction, λ1) and radial (transverse diffusion component, [(λ2+λ3)/2] diffusivity values were computed for clusters showing a significant FA change (increase or decrease) between the HA residents and SL controls. Data were analyzed using SPSS. ANOVA statistic identified the differences between the HA and SL groups. Data were presented as mean ± SD. Statistical significance was set at *p*<0.05.
